# MBBS Students’ Perspectives on the Benefits and Challenges of the Family Adoption Program: A Mixed-Methods Cross-Sectional Study of Medical Colleges in Uttar Pradesh, India

**DOI:** 10.7759/cureus.98387

**Published:** 2025-12-03

**Authors:** Srikrishna Sulgodu Ramachandra, Imran Ahmed Khan, Richa Mishra, Priya Dubey, Harish C Tiwari, Arun K Srivastava, Umesh K Verma

**Affiliations:** 1 Community Medicine, Keshav Memorial Charity (KMC) Medical College and Hospital, Maharajganj, IND; 2 Community Medicine, Mahamaya Rajkiya Allopathic Medical College, Ambedkar Nagar, IND; 3 Community Medicine, Baba Raghav Das (BRD) Medical College, Gorakhpur, Gorakhpur, IND

**Keywords:** community health services, competency-based medical education, family adoption program, medical student’s perspective, undergraduate medical education

## Abstract

Introduction

The Family Adoption Program (FAP) was introduced in medical colleges across India. It was done to provide early clinical exposure and incorporate a sense of social responsibility and empathy among medical students. The program involves students adopting families in the community to understand their health, socio-economic, and living conditions while facilitating primary healthcare.

Objectives

This study aims to assess the knowledge of first-year Bachelor of Medicine, Bachelor of Surgery (MBBS) students from three medical colleges about the objectives and overall expected outcome of FAP; to identify perceived challenges and benefits of the FAP to the medical students and to the adopted families; and to gather suggestions for the improvement of FAP.

Materials and methods

This is designed as a cross-sectional descriptive, mixed-methods approach study. The study population included first-year MBBS students (batch of 2024-25) from three medical colleges in Uttar Pradesh state of India. The principal investigator’s college was selected based on convenience sampling and feasibility, and two other colleges were randomly selected for the study. The sample size for the quantitative study is expected to include all first-year MBBS students (400 students) from the three medical colleges. A pretested semi-structured questionnaire was administered online using Google Forms (Google, Mountain View, CA, US) to collect the quantitative data. The qualitative study was done using focus group discussion (FGD). Two FGDs with 10 participants each were conducted in each of the included medical colleges to understand the qualitative aspects of the FAP.

Results

About 81% of students participated in the study by filling out the Google Forms, and 60 students participated in the FGD. Around 90% of the students mentioned skill development and making students understand rural life and health problems as objectives of the FAP. Around 4% of students mentioned the dual purpose of FAP, which included skill development of the students and health improvement of the adopted families. The students mentioned learning communication skills, understanding social determinants of health, teamwork and leadership skills, and medical knowledge application as learning from FAP. The challenges highlighted by students included language barriers, communication gaps, logistics and transportation, inability to get cooperation from family members, and non-availability of family members. Health education, screening for diseases, early referral, and overall improvement of the health status of adopted families were perceived as benefits to the adopted families.

Conclusion

Overall, the medical students’ knowledge and perspective seem to be very positive toward the FAP. FAP, incorporated into the undergraduate medical education curriculum, seems to be beneficial to all the stakeholders involved. FAP helps in achieving holistic learning for the medical students and also helps in the overall improvement of the health of the adopted families and, in turn, the community.

## Introduction

The Family Adoption Program (FAP) has been introduced in all medical colleges across India, as per the National Medical Commission Undergraduate Medical Education Board (NMC-UMEB) Competency-Based Medical Education (CBME) guidelines [1,2]. This is done to provide early clinical exposure (ECE) and incorporate a sense of social responsibility among undergraduate medical students [3]. The program begins in their first professional year and continues till their third professional year as part of the curriculum of Community Medicine and is executed by the same department [1,2]. The program involves students adopting families in the community to understand their health, socio-economic, and environmental conditions while facilitating primary healthcare [4,5]. This initiative aims to provide experiential learning opportunities to Indian Medical Graduates (IMGs) toward community-based healthcare and bridge the gap between medical education and community health needs [6]. It is expected to improve the communication skills of the students, which is an essential skill in medical practice. It helps students learn the right attitude and empathy toward the families that they have adopted and the community in general. Along with offering community-based training for healthcare professionals, the FAP promotes a community-oriented approach by improving healthcare access for impoverished families [7]. This aligns with India’s commitment to achieving Universal Health Coverage (UHC) by 2030 [8] and toward achieving Sustainable Development Goals (SDGs) [9].

FAP was conceptualized upon the “Sewagram Model” [10] by the Mahatma Gandhi Institute of Medical Sciences (MGIMS), Sewagram. The FAP program was introduced in August 2019 to be implemented for Bachelor of Medicine, Bachelor of Surgery (MBBS) students from the 2021-2022 batch as part of the new CBME curriculum. This was a conscious effort by the NMC (erstwhile Medical Council of India (MCI)) to ensure that the medical students receive a community-based, hands-on learning experience from their very induction stages into the medical school [11,12]. While the program, as a concept, is innovative and potentially transformative, there will always be challenges in the rollout and implementation of any new program. On another note, it is essential to understand how first-year MBBS students perceive this early exposure to community health settings. Since FAP is a relatively new initiative, there are just a handful of studies that dwell on the perspectives, benefits, and challenges of FAP. Hence, there is a need to build more evidence on the FAP.

This mixed-methods study was conducted to assess the perceived understanding and experience of first-year MBBS students on the FAP. The study is expected to generate feedback and inputs that can help improve the design and implementation and thereby strengthen the program for the future batches of MBBS. The objectives of this study were (1) to assess first-year MBBS students’ knowledge of the objectives and expected outcomes of the FAP, (2) to identify the perceived benefits and challenges of the FAP to themselves and to the adopted families, and (3) to gather suggestions for improvement of the FAP.

## Materials and methods

This is designed as a cross-sectional descriptive, mixed-methods approach study. The study population included first-year MBBS students (batch of 2024-25) from three medical colleges in Uttar Pradesh state of India. The study included Baba Raghav Das (BRD) Medical College, Gorakhpur; Keshav Memorial Charity (KMC) Medical College, Maharajganj; and Mahamaya Rajkiya Allopathic (MRA) Medical College, Ambedkar Nagar. Both BRD Medical College and KMC Medical College have 150 students each in the first year of MBBS, and MRA Medical College has 100 students in the first year of MBBS. Both BRD and MRA are government medical colleges, and KMC is a medical college set up in a Public-Private Partnership (PPP) mode. The principal investigator’s college was selected based on convenience sampling and feasibility, and two other colleges were randomly selected for the study. After randomly selecting two colleges, we contacted the concerned departments in those colleges and requested them to be part of this study, to which they agreed. Although simple random sampling of all medical colleges in Uttar Pradesh was initially considered, logistical constraints (travel distance, existing collaboration, and willingness of the Community Medicine departments to facilitate the study) led to purposive selection of these three institutions.

Ethics approval for the conduct of this study was obtained from the Institutional Ethics Committee (IEC) of the BRD Medical College (S. No. 244/IHEC/2025). The sample size for the quantitative study is expected to include all first-year students of MBBS (2024-25 batch: 400 students) from the three medical colleges mentioned above. However, a rejection rate of 10% was expected, as involvement in the study was voluntary for the students. A census approach was chosen because the population is finite, relatively small, and highly homogeneous (first-year students exposed to the same NMC-mandated FAP curriculum), making a census more feasible and informative than sampling a fraction.

The qualitative study was done using focus group discussion (FGD). Two FGDs (one FGD with exclusively male students and one with exclusively female students), having 10 participants per FGD, were conducted in each of the included medical colleges to understand the qualitative aspects of the FAP. Therefore, this amounted to a sample size of 60 students.

Data collection tools

A pretested semi-structured questionnaire was administered online using Google Forms (Google, Mountain View, CA, US) to collect the quantitative data. The questionnaire was developed by the authors based on (a) NMC guidelines on FAP, (b) published studies on FAP perceptions, and (c) expert consultation with five senior faculty members of Community Medicine departments. Content validity was ensured through this process and pilot testing on 20 students. Participants were presented with a digital informed consent form at the beginning of the online questionnaire. Only those who consented proceeded to answer the survey. Responses were collected anonymously; no names, roll numbers, emails, or internet protocol (IP) addresses were recorded. The faculty left the classroom during survey completion to prevent perceived coercion. The quantitative data were collected with the help of medico-social workers (MSWs) in the department of Community Medicine. Only the principal investigator had access to the response spreadsheet. Data were downloaded immediately after closure of the form, stored on a password-protected server, and will be deleted five years after publication. The study followed research ethics standards and the Declaration of Helsinki.

A topic guide was used for the FGDs (qualitative data). Potential participants who were identified by the authors as vocal, outspoken, and likely to contribute to the objectives of the discussion were approached and included in the FGD. The FGDs were conducted in person. The proceedings of the discussion were recorded after taking verbal consent from all the concerned participants during the recording itself. They were also informed on record of their right to exit from the FGD at any stage of the discussion. The FGDs were conducted on the basis of a loosely structured topic guide, and newer inputs were probed. All the recordings of the FGDs were transcribed, coded, and categorized under specific themes. The coding and thematic analysis were done manually by the first author, and consensus was obtained on the codes and themes with the other authors.

For the pilot testing and validation of the tools (semi-structured questionnaire and topic guide - included as Appendices 1 and 2), a pilot test of the tools was conducted in order to ensure that the questions were understood by the participants, for language corrections, for standardizing the tool, and also for ensuring that the responses obtained were in line with the expected outcome. The Google Form was pilot-tested on 20 medical students (who did not form part of the actual sample size of the study), and also, one FGD was conducted. Based on the results of this pilot testing, necessary corrections and changes were made in the tools.

The duration of the study was three months (July 1, 2025, to September 30, 2025). Data were entered in Microsoft Excel (Microsoft Corp., Redmond, WA, US) and analyzed using IBM SPSS version 25 (IBM Corp., Armonk, NY, US). Quantitative descriptive statistics were computed as frequencies and percentages. Categorical associations were tested with Pearson’s chi-squared test. A p-value < 0.05 was considered significant. Qualitative data were analyzed using content analysis (based on themes).

## Results

The results of the study are given in two sections: Section A: results based on quantitative data, and Section B: results based on qualitative data.

Section A: results based on quantitative data

**Figure 1:**
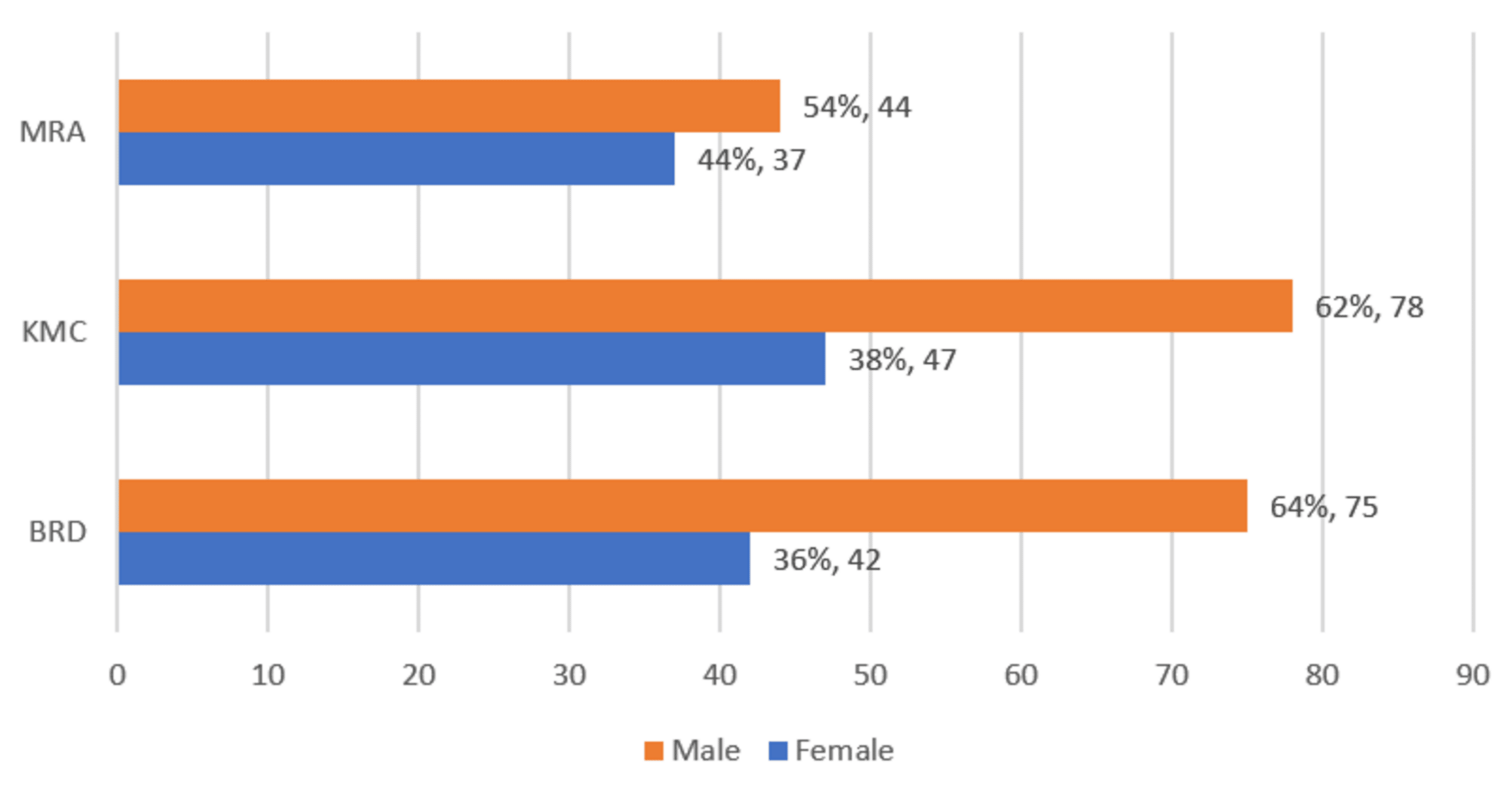
College-wise and gender-wise distribution of medical students in the study BRD: Baba Raghav Das; KMC: Keshav Memorial Charity; MRA: Mahamaya Rajkiya Allopathic

Out of the 400 students from the three medical colleges, a total of 323 students participated in the survey (80.75% response rate) by filling in the required information in the Google Forms. The age of the students who participated in the study ranged from 17 to 29 years, with 80% (n = 258) of the students aged between 19 and 23 years. The study sample comprised 61% (n = 197) male students and 39% (n = 126) female students, with a total of 323 medical students (Figure 1).

**Figure 2:**
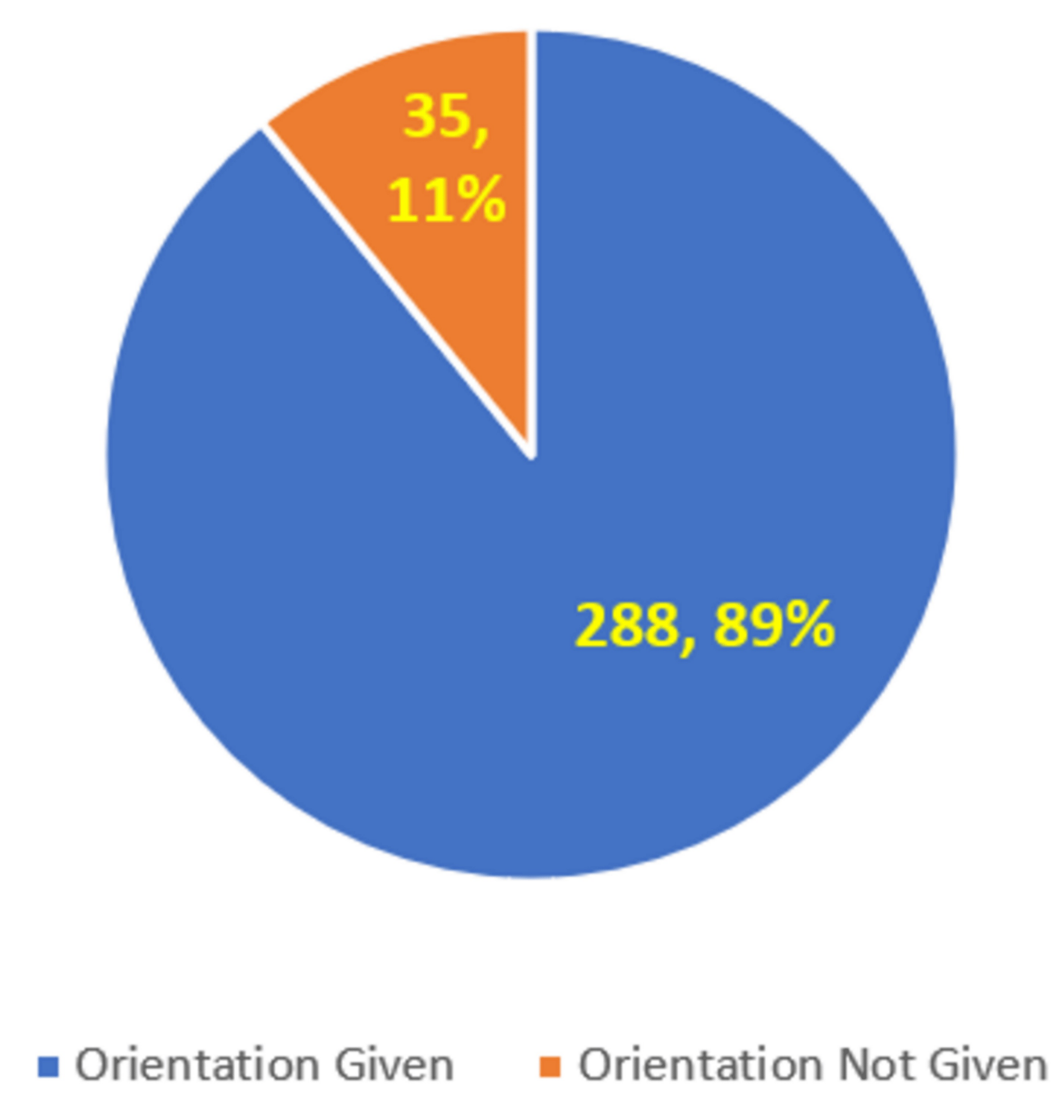
Orientation given before visiting the community for the FAP FAP: Family Adoption Program

A little more than 89% of the students (n = 288) mentioned that an orientation was given prior to starting the FAP, and around 11% (n = 35) mentioned that no orientation was given before the start of the FAP (Figure 2).

Students from all three medical colleges mentioned that they were provided with hard copies of the logbook for documenting the details from the visits. The medical students’ perception regarding the objectives of FAP is given in Table 1. About 90% (n = 290) of the medical students mentioned that skill development and making the students understand the health and living conditions of the rural families, and also rural life, were the main objectives of the FAP. Around 6.5% (n = 21) of the medical students mentioned that health improvement of the adopted families was the main objective of FAP. Only around 4% of the medical students mentioned that the development of skills of the medical student, understanding of rural life, health and living conditions, and also health improvement of the adopted families were the key objectives of the FAP.

**Table 1:** Medical students’ perception regarding the objectives of FAP FAP: Family Adoption Program

Main objectives of FAP (multiple responses were allowed)	Frequency	Percentage
Skill development (communication skill, leadership skill, teamwork, imparting health education, empathy, and clinical skills - early clinical exposure)	115	35.60%
Students’ understanding of rural health status, financial and living conditions of rural families, and early community exposure	98	30.34%
Students’ understanding of rural health status, financial and living conditions, early clinical and community exposure, and skill development	77	23.84%
Health improvement of the adopted families	21	6.50%
Skill development (communication skill, leadership skill, teamwork, imparting health education, empathy, and clinical skills), and health improvement of the adopted families	7	2.17%
Students’ understanding of rural health status, financial and living conditions, early clinical and community exposure, skill development, and health improvement of the adopted families	5	1.55%
Grand total	N = 323	100%

**Figure 3:**
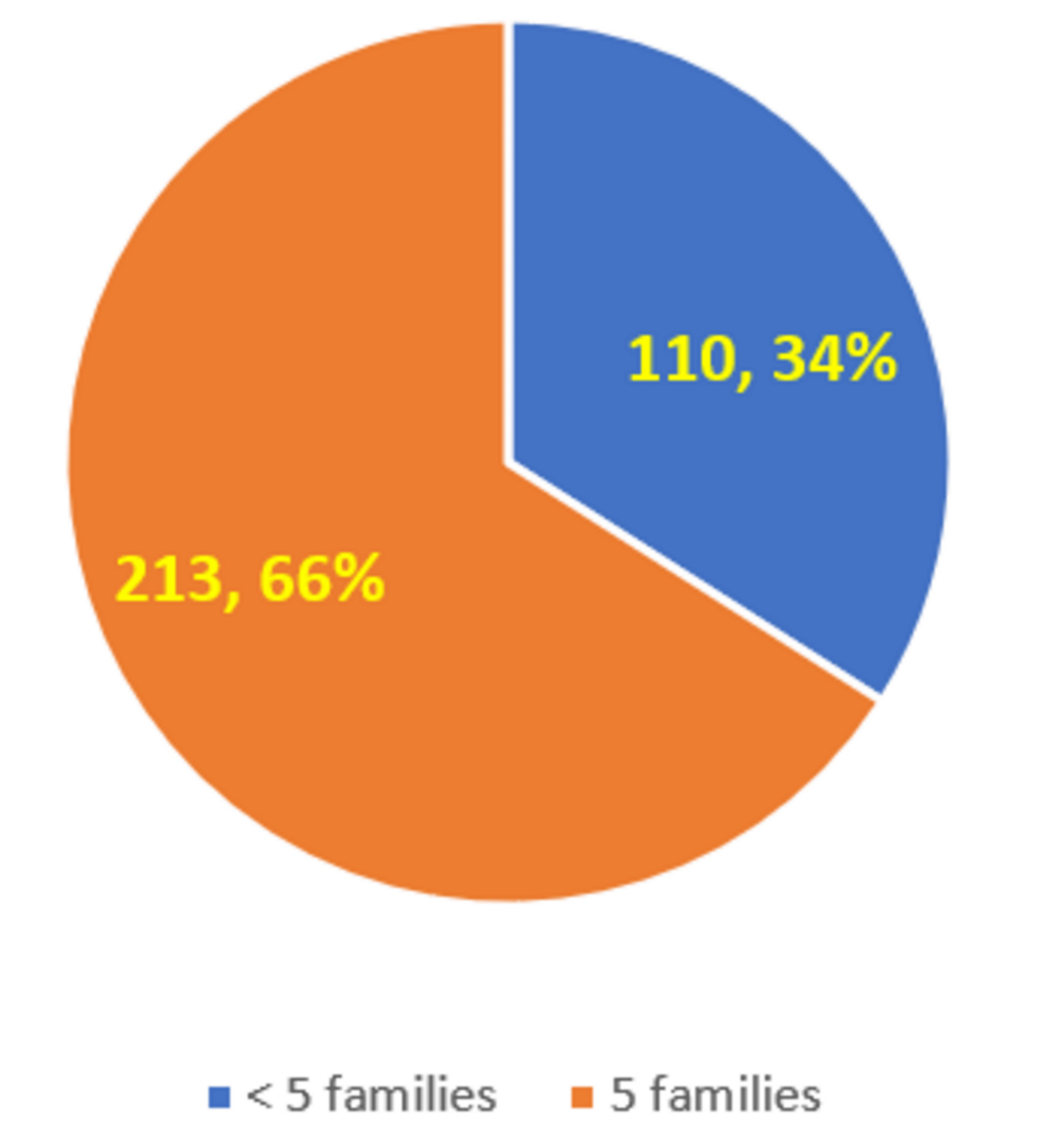
Medical students’ knowledge of the number of families to be adopted by each student as per the NMC guideline NMC: National Medical Commission

Figure 3 shows that around 66% (n = 213) of the students mentioned that each student is supposed to adopt five families under the FAP as per NMC guidelines, whereas around 34% (n = 110) of the students mentioned that each student is supposed to adopt less than five families (this included options of adopting three, two, and one family).

**Figure 4:**
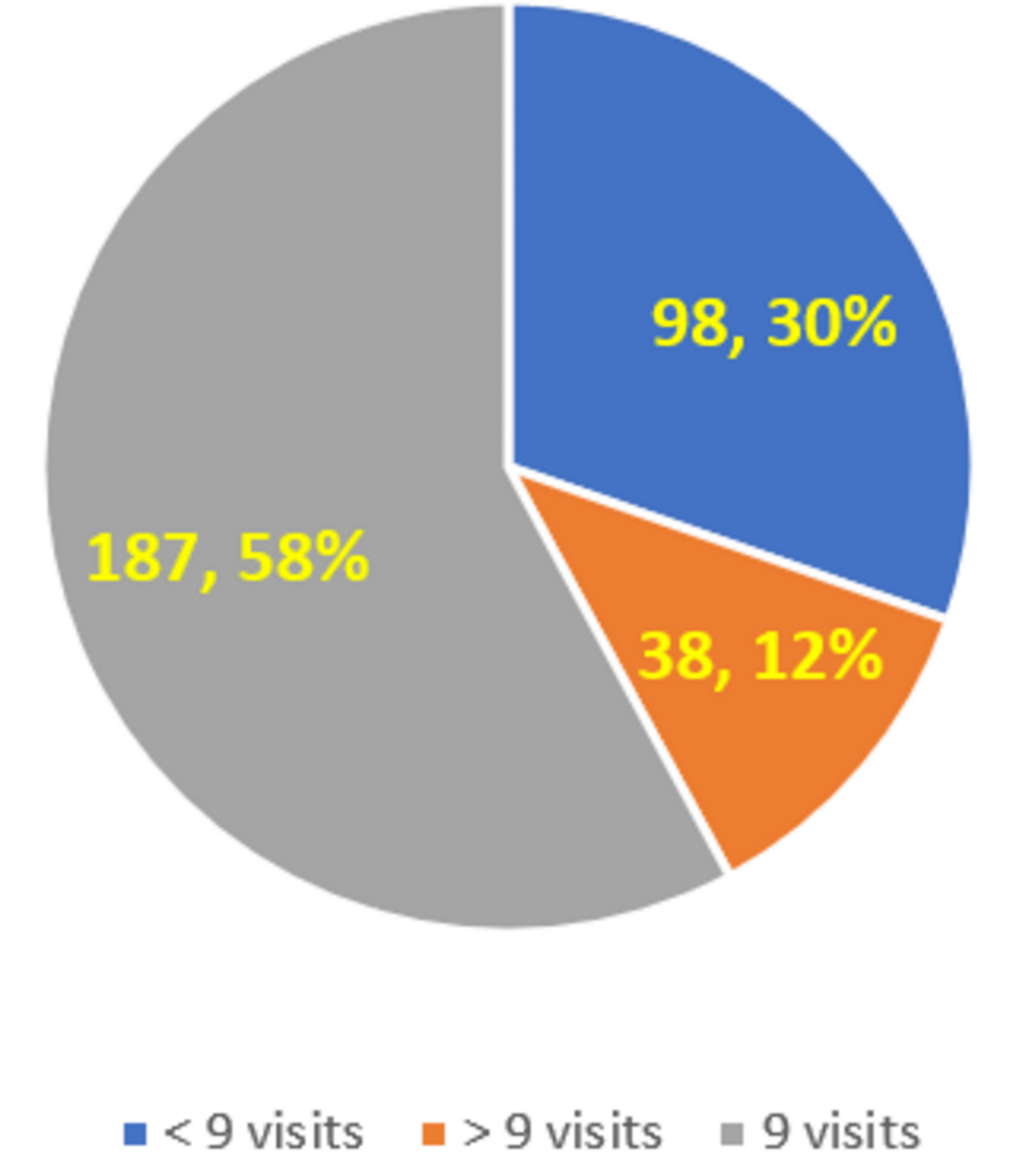
Medical students’ knowledge about the number of FAP visits in the 1st professional year period (as per NMC guideline) NMC: National Medical Commission; FAP: Family Adoption Program

Around 58% (n = 187) of students mentioned that there should be a total of nine visits in the first professional year for the FAP, as per the NMC guideline. Around 30% (n = 98) of the students mentioned that there should be less than nine visits in the first professional year, and around 12% (n = 38) of the students mentioned that there should be more than nine visits (Figure 4).

One hundred and seventy-nine (55.4%) students mentioned that they were able to gain communication skills, teamwork, and leadership skills; were able to understand the social determinants of health; and also apply some of the basic medical knowledge that they learned as part of orientation to FAP. About 20% (n = 62) of the students mentioned that they learned any one of the above skills (Table 2).

**Table 2:** Learnings from interacting with adopted families in the FAP FAP: Family Adoption Program

Learned skills (multiple responses were allowed)	Frequency	Percentage
Communication skills, understanding social determinants of health, and medical knowledge application	29	8.98%
Communication skills, understanding social determinants of health, teamwork, leadership skills, and medical knowledge application	179	55.42%
Communication skills, understanding social determinants of health, teamwork skills, and medical knowledge application	35	10.84%
Understanding social determinants of health	18	5.57%
Mentioned learning any one of the above skills	62	19.20%
Total	323	100.00%

Challenges faced during FAP are compiled in Table 3. Thirty-two percent (n = 103) of the students mentioned that language barriers were the main challenge they encountered during their interactions with adopted families in the FAP. Twenty-eight percent (n = 88) of the students mentioned that “inability to get cooperation from families” was a challenge they faced during FAP. Fifteen percent mentioned that logistics and transportation were a challenge, and 10 (2.5%) mentioned the non-availability of family members in the adopted families as a challenge.

**Table 3:** Challenges faced during FAP FAP: Family Adoption Program

Challenges faced (multiple responses were allowed)	Frequency	% of total
Language barrier	103	32%
Inability to get cooperation from family	63	20%
Communication gap	62	19%
Inability to get cooperation from family; language barrier	25	8%
No challenges faced	13	3.5%
Logistics - transportation	47	15%
Non-availability of family members	10	2.5%
Total	323	100%

**Figure 5:**
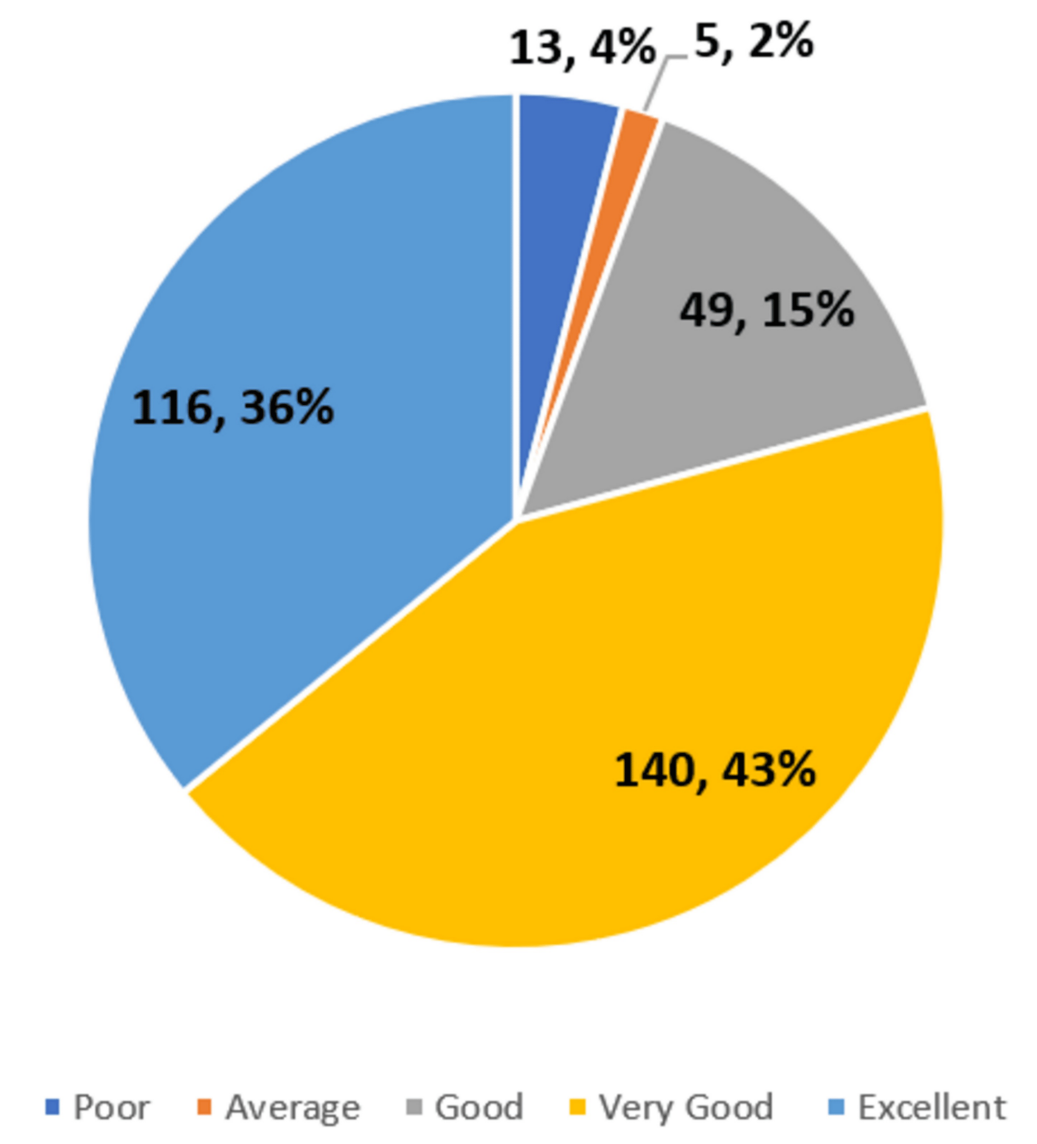
Overall rating of experience with FAP FAP: Family Adoption Program

When asked about a rating on a scale of 1 to 5 (1 being poor and 5 being excellent) about the overall experience of FAP in their first professional year, 36% (116/323) of the students have mentioned excellent, 43% (n = 140) of the students have mentioned very good, and 15% (n = 49) of the students have mentioned good. Around 6% (n = 18) of students have mentioned their overall experience as average or below (Figure 5).

Fifty-eight percent (n = 186) of the students mentioned that health education and awareness on various health-related aspects, including basic sanitation and hygiene, the importance of a clean surrounding environment, clean drinking water, and immunization, were perceived as benefits of the FAP to the adopted families. Thirty-one percent (n = 101) of students mentioned screening for diseases, early referral, some medications, and health education along with benefits to the adopted families as part of FAP. Eleven percent (n = 34) of students mentioned that screening for common non-communicable diseases and anthropometric measurements were perceived benefits to the adopted families under FAP (Table 4).

**Table 4:** Perceived benefits of FAP to the adopted families FAP: Family Adoption Program; BMI: body mass index

Perceived benefits (multiple responses were allowed)	Frequency	% of total
Health education on basic sanitation and hygiene, the built environment, keeping the house and surroundings clean, the importance of clean drinking water, and immunization	186	58%
Health education on basic sanitation and hygiene, the built environment, keeping house surroundings clean, the importance of clean drinking water, and immunization; early referral	4	1%
Health education on basic sanitation and hygiene, the built environment, keeping the house and surroundings clean, the importance of clean drinking water, and immunization; screening for diseases	7	2%
Health education on basic sanitation and hygiene, built environment, keeping house surroundings clean, the importance of clean drinking water, immunization, and screening for diseases; early referral	34	11%
Medical check-ups, medication, and early referral as and when needed	56	17%
Screening for hypertension, diabetes, BMI, anemia, and anthropometric measurements	36	11%
Grand total	323	100%

The data were analyzed for the association between challenges faced during FAP versus gender to see if there were any specific, unique challenges for a particular gender; there was no significant relationship between gender and challenges faced during FAP. The data were also analyzed for the association between challenges faced during FAP versus medical colleges based in rural areas and medical colleges based in urban areas. There was no significant relationship between the location of the medical college and challenges faced during FAP (Table 5).

**Table 5:** Association between challenges faced vs. gender and geographical location of the college

Variables	Chi-squared value	p-value
Challenges faced versus gender	8.184	0.225
Challenges faced versus geographical location of the medical college (urban/rural)	8.881	0.180

Section B: results based on qualitative data

Two FGDs (one exclusively with male medical students and one exclusively with female medical students) were conducted in each of the three medical colleges. Ten medical students participated in each FGD, for a total of 60 participants. The duration of each FGD lasted 40 to 45 minutes.

Themes

Initially, the topic guide for the FGD was prepared based on the following themes: knowledge about FAP and its objectives, implementation of FAP and the challenges faced, perceived benefits of FAP to medical students, perceived benefits of FAP to adopted families and the community, and suggestions to improve FAP. In addition to the above, during the data analysis, the following themes emerged: community response to FAP, additional support from the medical college that would enhance FAP, and the roles and responsibilities of medical students and faculty in improving the overall quality of FAP. The participants of the FGDs had mixed responses in relation to the various themes discussed.

Theme 1: knowledge about FAP and its objectives:

“FAP visits act as a rapport-building exercise for us and help in acquiring good communication skills and leadership skills.”

“FAP provides early community exposure and also early clinical exposure. It gives us an opportunity to implement and experience what we learn in the classroom.”

“It helps to improve the health and well-being of the adopted families.”

“The Gram Pradhan and the adopted families are happy, as they are screened for non-communicable diseases, and referral to the hospitals is done.”

Theme 2: implementation of FAP and challenges faced: Some of the key challenges mentioned included logistics issues (transportation), actual time spent with the families, language barriers, not being able to get cooperation from the families, non-availability of all the family members during the visit, and weather conditions.

“We spent more time waiting for our transportation and travel than the actual time spent with the adopted families.”

“The local language in this region is Bhojpuri, and in the majority of the rural areas, women especially speak only this language, and for us students who have come from outside of Uttar Pradesh, communicating with these community members becomes very difficult.”

“At times, a single female is available in the family, and the family is allotted to a male student; it becomes difficult. Similarly, in some families, when only male members are available and a female student visits that family, it becomes very difficult to interact and collect data.”

“High expectations from the family-many families initially expect that when a person with a white coat comes, they would provide some investigations and treatment (medicines) for their ailments and not just collect data and provide health education.”

“The moment I said I am from this college and hospital, they closed the door on me.”

“This area has extremes of climate: extreme cold in winters and severe heat during summers-so the visits should be planned in such a way that the weather is conducive …”

Theme 3: perceived benefits of FAP to the medical students:

“Early community exposure and early clinical exposure will help us become more empathetic and understanding of the various ways in which people behave in the community, and that is a lot of learning ….”

“Some of us had never visited a rural area and never seen life in a rural setting, problems in a rural area… So, this was a good opportunity for us to witness the same …”

“Along with the learning aspects associated with FAP, the village visits act as an outing and as a stress buster, since during the first professional year of medical school, we are not given many opportunities to go out of the campus.”

Theme 4: perceived benefits of FAP to the adopted families and community:

“As part of the FAP, we gave health education on basic sanitation and hygiene practices, built environment (cross ventilation and lighting), keeping the house surroundings clean, the importance of clean drinking water, the importance of regular health and wellness visits to a health care facility …”

“We informed them on the importance of antenatal care, institutional delivery, exclusive breastfeeding, and vaccination for the newborn and under-fives …”

“The importance of having toilets in the houses, harmful effects of open-air defecation, oral hygiene, hand hygiene, the importance of elementary and collegiate education, etc., were told to my adopted families.”

“We gave referral slips to families wherein people had chronic/acute illnesses, and they were referred to our medical college hospital for further management.”

“We screened them for diabetes mellitus and hypertension, and family members with these problems were referred to our medical college hospital, so the families were grateful to us for that …”​​​​​​​

Theme 5: suggestions to improve FAP:

“We need more visits to the community and the adopted families for a better understanding and follow-up.”

“Structured orientation, more involvement from students and faculty alike, a designated mentor for each student or a group of students, and feedback sessions by senior faculty are needed.”

“In this digital era, digital documentation of the findings rather than hard copies of the logbook would be better.”

“A uniform template of the logbook with core requirements applicable for all medical colleges and flexibility to include local nuances.”

“More mentorship and training before we actually go to the adopted families.”

“Transportation and logistics issues to be improved.”

“Some basic free medicines should be provided to the adopted families as part of the FAP.”

“Regular feedback and assessment of the visits would strengthen the program.”

“The families and the community should be made more aware of the program before we go to the actual visits.”

“At least 2 students together should be allotted a family or set of families.”

“Integrate FAP with clinical learning and research.”​​​​​​​

Theme 6: communities’ response to FAP:

“Some of the families received us very well and gave us all the information needed, whereas some households acted indifferent …”

“It took us a couple of visits to make the families understand the purpose of our visit and clarify their misconceptions.”

“After a good rapport was built with one of my adopted families, they even served tea and snacks to me.”

“Some families have read about scams and frauds, and hence, neither wanted any information from anyone nor wanted to give any information about themselves ….”

“When a staff member or faculty member accompanies us to the family, they talk very nicely and give all the necessary information, but when we go by ourselves, they don’t bother much ….”​​​​​​​

Theme 7: additional support from the medical college that would make FAP better:

“Timely provision of vehicles for transportation and other logistics will strengthen the FAP …”

“More portable instruments (for anthropometric measurements, checking blood pressure and blood sugar levels) will be very helpful …”

“More involvement and guidance from faculty will help us.”

“A few more investigations and free medicines during the FAP camp will help in strengthening the program for future visits and next batches …”​​​​​​​

Theme 8: roles and responsibilities of medical students and faculty to improve the overall quality of FAP:

“Proper orientation and guidance related to the village map, etc., should be given in the lecture classes just before the FAP visit so that it is easy for us to go to the field confidently ….”

“More involvement by senior faculty from the Community Medicine department in the field will help us understand things better.”

“We focus more on the 1st professional year subjects and don’t take Community Medicine seriously in our 1st year, so we need to change this attitude …”​​​​​​​

## Discussion

The discussion part is given in two sections: Section A: discussion based on the results of the quantitative data, and Section B: discussion based on the results of the qualitative data.

Section A: discussion based on the results of the quantitative data

The overall response rate in our study was 80.75% (288/323). Usually in online surveys, the response rate is anywhere between 25% and 30%, based on various factors [13-15]. However, since this was a study in an academic setting, one of the good practice methods to achieve a high response rate in an online survey in an academic setting is to share the online Google Forms in a classroom, explain the rationale of the study to the potential participants, inform them that participation is voluntary, and request them to fill in the online survey. By following this method, we were able to achieve more than 80% response rates in all three centers where we conducted the study.

As seen in Figure 2, the majority (89%, n = 288) of the survey participants mentioned that an orientation was given prior to starting the FAP in all three medical colleges. It is very likely that the remaining 11% (n = 35) of students who mentioned “Orientation not given” were probably absent on the particular day/s when the orientation was given.

The majority of the students (around 90%, n = 290) perceive that the main objective of FAP is to ensure that the students enhance their communication skills and leadership skills and also get a good understanding of rural community settings, the life and health status of community members in the rural area, and also the health problems in a rural community. Six and a half percent of students mentioned that health improvement of the communities is the only objective. Only around 3.7% (n = 12) of the students have a holistic understanding of the objectives of FAP, which includes both skill development and early community and clinical exposure for students, and also ensuring that the health of the rural communities is improved. Several other studies published in the recent past have shown similar results [16-19]. Overall, our study denotes that the students have a fairly good understanding of the objectives of the FAP.

In our view, it is important for the medical colleges to give a very good orientation of the objectives of the FAP before the students go to the field, and that they understand the dual purpose of FAP. This is absolutely essential to achieve one of the targets of UHC by 2030 [8], which is about ensuring health education, promoting health, screening for both communicable and non-communicable diseases, and also educating the communities about health and well-being. This would definitely go a long way in India in achieving the SDG 3 - Good Health and Well-Being [9].

Around one-third (32%, n = 103) of the students were not able to correctly recollect the number of families to be adopted by each student. This seems to be a bit of concern, as this is one of the first information that is to be made clear to the students during the orientation of the FAP itself. Therefore, as long as there is no clarity about this basic fact, it is likely that the students would not have followed up on the next steps as well. It is important that the Community Medicine department and the concerned college authorities emphasize this in future batches. As per the NMC guideline, there should be a total of nine FAP visits (eight to nine three-hour visits totaling 24 teaching hours under the CBME curriculum) [1,2] to the villages in the first professional year. As shown in Figure 4, this was rightly mentioned by around 58% (n = 187) of the students who were interviewed. In many colleges, the number of visits has crossed nine visits - as in several situations, when the students have not completed data collection in the stipulated nine visits, more visits were scheduled so that they can go back to their adopted families and complete the data collection. Therefore, it is very likely that the 12% (n = 38) of the students who responded with more than nine visits in the first professional year have said it based on their personal experience. However, it is a bit worrisome, wherein 30% (n = 98) of the students have mentioned that there should be less than nine visits in the first professional year. This denotes a lack of understanding among these students of the overall FAP, and this knowledge level issue should be addressed in future batches. The concerned colleges and the Community Medicine departments should emphasize the importance of the community visits as part of the FAP and should ensure that students go to the field as part of the FAP. We could not find relevant studies that have looked at the knowledge of students about the number of families to be adopted by each medical student under the FAP program and also relevant studies that have looked at the knowledge of students about the total number of visits in the first professional year of MBBS.

For the variables in Figures 3, 4, a college-wise and gender-wise breakup of these variables is purposefully not included as part of this manuscript, as the whole idea was not to compare between medical colleges and highlight shortcomings in a particular medical college. Our motto was to identify shortcomings in general and address them in future batches of MBBS.

The majority of the students were very positive about the FAP and mentioned that they learned various skills, including communication skills, leadership skills, empathy, and teamwork. The students also mentioned learning the social determinants of health, which included poverty, education, and accessibility and availability of health care. They also mentioned applying the medical knowledge that they gained during the orientation program. This included history-taking, measuring pulse and blood pressure, anthropometric measurements (height, weight, mid-upper arm circumference, skin thickness, and calculating body mass index), and looking for pallor and icterus. Our results are in line with several other studies that were published from various medical colleges across India [18,19].

As shown in Table 3, the major challenges faced by the medical students included language barriers, communication issues, not being able to get cooperation from families, and logistics (transportation). It is obvious that when students from different states join a particular medical college, there are bound to be some language barriers and communication issues. These results of ours are in concurrence with results from other similar studies [15-18]. The only solution for this is that the students learn some basics of the local vernacular, as it would be needed for them in the clinical practice as well (second, third, and final professional years). Also, one of the colleges that was part of the survey, being a relatively new college, has no dedicated vehicle for the Community Medicine department. These issues will obviously be sorted out sooner rather than later. The issue of “inability to get cooperation from families” needs to be addressed by having a strong prior outreach activity by the Community Medicine department (MSWs, staff, and faculty) along with the close involvement of Accredited Social Health Activists (ASHAs), Anganwadi Workers (AWWs), Auxiliary Nurse Midwives (ANMs), and Gram Pradhan (village head). Students should also be oriented about sympathy, empathy, and etiquette before the family visits.

As seen in Figure 5, 94% (n = 304) of the medical students had a good to excellent overall experience of the FAP. This is absolutely a positive vibe and shows that the FAP is definitely a good initiative in spite of all the challenges and shortcomings that are to be addressed. Health education and awareness, screening for non-communicable diseases, and early referral were perceived as the benefits of FAP to the adopted families. These results are in concurrence with some of the previously published studies [16-18]. In addition to this, whenever a camp was conducted as part of the FAP, essential investigations, treatment, and medications were given free of cost to the patients from the adopted families.

Section B: discussion based on the results of the qualitative data

As highlighted in the Results section, the participants of the FGDs had mixed responses on the themes that were discussed. Around half of the participants had a fairly good understanding of the dual purpose of the FAP, which is knowledge and skill development of the students and also health improvement of the adopted families and, in turn, the community. For the remaining half, they had to be reminded of the dual purpose of the FAP, but they quickly agreed to the same. Here, we feel that it is important for the faculty from the Community Medicine department to emphasize and re-emphasize the dual purpose of the FAP during the orientation itself and also at different points during the course of the program. Similar results have been observed in a couple of previous studies [15,17-20].

Our results concur with the results of previous studies wherein the various challenges discussed were commonly mentioned in other studies as well [18,20-23]. One of the medical colleges that was part of the study was a relatively new medical college - only one batch of medical students has been enrolled. Therefore, despite having good faculty and infrastructure at this college, there is no dedicated vehicle (college bus) for the Community Medicine department, which has made arranging transport a challenge. The authors anticipate that these logistics issues will be resolved sooner rather than later.

For challenges related to language barriers and also for situations wherein there is only a female member in the family, etc., the authors recommend that it would be helpful to pair up students in twos or threes and allocate a set of families to them; therefore, it is likely that at least one of them knows the local language, and also, if at least two students (both male and female) visit the family, then the issues of a single male/female in the family could be addressed.

For challenges related to “expectations from the family,” it is important for the Community Medicine department (MSWs and faculty) to have a good rapport with the ASHAs and AWWs and also with the Gram Pradhan (head of the village) so that they inform the concerned families about the motto of the FAP and also what to expect and what not to expect from the FAP visits. A good outreach activity before the start of the FAP is of utmost importance for this reason.

At times, the pre-existing brand image of an institution - for instance, if the cost of services (investigations and treatment) is felt by the community to be on the higher side, or if a particular family has had a bad experience from a particular hospital - may result in family members and community members in that particular village being less interested in interacting with the students from that concerned institution. Therefore, it is also important for the institutions to work toward building a good brand image for themselves.

Health education is done by the ASHAs, ANMs, and AWWs on a regular basis, but these additional visits by medical students as part of the FAP act as a booster dose and also give an opportunity for the members of the adopted families to have an intermediary contact point whom they can reach out to in times of any health issues/awareness issues. Each of these activities would, in its own little way, lead toward achieving the goal of UHC by 2030 and thereby help in achieving the broader SDGs for India [8,9].

There were some good suggestions that came out of the FGDs, which included having a uniform logbook format for all medical colleges by the NMC and also having a digital version of the logbook. A minimum of two students to be allotted a set of families was also a good suggestion, and the authors endorse the same. Enhanced interest and commitment from both faculty and students alike will go a long way in strengthening and sustaining the FAP. Emphasis on extensive outreach activity about the FAP is very important and is to be done in coordination and support of Gram Pradhan, ASHAs, ANMs, and AWWs.

The study has a few limitations. The inclusion of only three medical colleges in Uttar Pradesh may limit generalizability to other regions. Self-reported data may introduce bias. The cross-sectional design precludes assessment of the long-term impact of FAP. FGD participants were purposively selected as vocal students, potentially biasing qualitative findings toward more articulate views.

## Conclusions

Overall, the medical students’ knowledge and perspective seem to be very positive toward the FAP. FAP incorporated into the undergraduate medical education curriculum seems to be beneficial to all the stakeholders involved. FAP helps in achieving holistic learning wherein medical students get a first-hand exposure, understanding, relevance, and interconnectedness of several of the SDGs and also UHC. However, the utility and long-term sustainability of the FAP depend on several factors, including the active involvement, commitment, and guidance of the faculty in the Community Medicine department; ensuring adequate logistics and good outreach prior to the start of FAP, and also performing other beneficial activities for the overall betterment of the community, such as cleanliness drives, plantation drives, specific health education initiatives, regular wellness and free health check-ups, and camps with provision of essential medication to the adopted families. If implemented well, the FAP can definitely make a significant difference in Indian medical education and also the lives of adopted families and the Indian rural community as a whole.

## Appendices

Appendix 1

Semi-structured Questionnaire on Medical Students’ Perceptions of Family Adoption Program

Section A: demographic details of the medical student

1. Age (in completed years):

2. Gender (male or female):

3. Urban/rural background:

4. Name of the medical college wherein currently studying:

a. Baba Raghav Das (BRD) Medical College, Gorakhpur, UP

b. Keshav Memorial Charity (KMC) Medical College & Hospital, Maharajganj, UP

c. Mahamaya Rajkiya Allopathic (MRA) Medical College, Ambedkar Nagar, UP

Section B: details related to orientation/training/preparation before going to the community

5. Was any orientation/training given to you before visiting the community for the FAP?

Yes/no

Skip: if no to Que. 5, please go to Que. 8.

6. If yes to Que. 5, what details were given to you (answer in 2 or 3 key points)?

7. How long was the training/orientation program?

a. 1 hour to few hours

b. 1 day

c. More than a day

Section C: documentation of findings of the FAP

8. Is any format/logbook provided to you to note down the findings of the visit?

Yes/no

Skip: if no to Que. 8, please go to Que. 10.

9. If yes to Que. 8, is it a digital version of the logbook or a hard copy?

a. Digital version

b. Hard copy of a logbook

Section D: supportive supervision/monitoring and feedback/guidance in relation to FAP

10. Is any guidance/feedback given to you by your teachers on the data you have collected?

Yes/no

11. If yes to Que. 10, who guides you? (Multiple responses allowed)

a. Professor

b. Associate Professor

c. Assistant Professor

d. Senior Resident

e. Junior Resident

12. If yes to Que. 10, what type of feedback/guidance is given to you? (Answer briefly in 2-3 points)

13. Is there a mentor designated to you for guiding you in the FAP work as part of the mentor-mentee program, as per NMC guidance?

Yes/no

Section E: student’s knowledge, perceptions, and experiences on FAP

14. What, according to you, are the main objectives of FAP? (Multiple responses allowed)

a. Skill development (communication skill, leadership skill, teamwork, imparting health education, empathy & clinical skills - early clinical exposure)

b. Student’s understanding of rural health status, financial & living conditions of rural families, early community exposure

c. Student’s understanding of rural health status, financial & living conditions, early clinical & community exposure, skill development

d. Health improvement of the adopted families

15. How many families are to be adopted by a student under the FAP as per the NMC guideline?

Ans.:

16. In total, how many visits of FAP are to be done by each student during the MBBS 1st year period?

Ans.:

17. What did you learn from interacting with families? (Multiple responses allowed)

a. Communication skills

b. Understanding social determinants of health

c. Teamwork skills

d. Leadership skills

e. Medical knowledge application

18. What challenges did you face during the FAP? (Multiple responses allowed)

a. Language barrier

b. Not able to get cooperation from family

c. Communication gap

d. Logistics, transportation

e. Non-availability of family members

f. No challenges faced

19. Do you feel that the FAP is helpful in your medical training?

a. Yes, go to Que. 20

b. No, go to Que. 21

c. Not sure

20. If yes to Que. 19, why and how?

21. If no to Que. 20, why?

22. What changes would you suggest to improve the FAP? (Write briefly in 2-3 bullet points)

23. How would you rate your overall experience with FAP?

a. Poor

b. Average

c. Good

d. Very good

e. Excellent

24. Would you recommend FAP to be continued for future batches?

a. Yes

b. No

c. Maybe

25. What, according to you, are the perceived benefits of the FAP to your adopted families? (Multiple responses allowed)

a. Health education (on basic sanitation and hygiene, built environment, keeping house surroundings clean, and importance of clean drinking water and immunization)

b. Early referral

c. Screening for diseases (NCDs, anemia, BMI, and anthropometric measurements)

d. Medical check-ups and medication

Appendix 2

Topic Guide: Medical Students’ Perceptions of the Family Adoption Program

1. What do you know about the Family Adoption Program (FAP)?

2. What, according to you, are the objectives of the FAP?

3. Describe the process of implementation of FAP in your medical college.

4. Did you face any challenges in the overall process of FAP? Discuss in detail.

5. What are the benefits that you perceive for yourselves as medical students from the FAP?

6. What, according to you, are the benefits to the community as a result of the FAP implementation?

7. What are some of the shortcomings in the current FAP, and how do you think they could be improved?

8. Any other suggestions for improving the FAP?

9. What, according to you, was the community’s response to the FAP?

10. What additional support from the medical college, do you think, would make the FAP better?

11. What actions from medical students and faculty can improve the overall quality of the FAP?
